# The Emergence of Coxsackievirus A16 Subgenotype B1c: A Key Driver of the Hand, Foot, and Mouth Disease Epidemic in Guangdong, China

**DOI:** 10.3390/v17020219

**Published:** 2025-02-03

**Authors:** Huiling Zeng, Biao Zeng, Lina Yi, Lin Qu, Jiadian Cao, Fen Yang, Haiyi Yang, Chunyan Xie, Yuxi Yan, Wenwen Deng, Shuling Li, Yingtao Zhang, Baisheng Li, Jing Lu, Hanri Zeng

**Affiliations:** 1 School of Public Health, Guangdong Pharmaceutical University, Guangzhou 510006, China; 13163764733@163.com (H.Z.); shuling200003@163.com (S.L.); 2 Guangdong Provincial Institution of Public Health, Guangdong Provincial Center for Disease Control and Prevention, Guangzhou 511430, China; linayi2009@live.cn (L.Y.); quinnyl@163.com (L.Q.); caojd6@mail2.sysu.edu.cn (J.C.); yanghy99@mail2.sysu.edu.cn (H.Y.); 18720795430@163.com (C.X.); yanyuxi0214@163.com (Y.Y.); dwwwendy@163.com (W.D.); 3 Guangdong Provincial Key Laboratory of Pathogen Detection for Emerging Infectious Disease Response, Guangdong Workstation for Emerging Infectious Disease Control and Prevention, Guangdong Provincial Center for Disease Control and Prevention, Guangzhou 511430, China; stevenzeng202305@163.com (B.Z.); yangfen0888@163.com (F.Y.); zhangyt9@mail2.sysu.edu.cn (Y.Z.); libsn@126.com (B.L.); 4 School of Public Health, Sun Yat-sen University, Guangzhou 510080, China; 5 School of Basic Medicine and Public Health, Jinan University, Guangzhou 510632, China; 6 School of Public Health, Southern Medical University, Guangzhou 510515, China

**Keywords:** coxsackievirus A16, B1c clade, evolution of genotype, HFMD

## Abstract

Background: In 2024, mainland China witnessed a significant upsurge in Hand, Foot, and Mouth Disease (HFMD) cases. Coxsackievirus A16 (CVA16) is one of the primary causative agents of HFMD. Long-term monitoring of theCVA16 infection rate and genotype changes is crucial for the prevention and control of HFMD. Methods: A total of 40,673 clinical specimens were collected from suspected HFMD cases in Guangdong province from 2018 to 2024, including rectal swabs (n = 27,954), throat swabs (n = 6791), stool (n = 5923), cerebrospinal fluid (n = 3), and herpes fluid (n = 2). A total of 24,410 samples were detected as EV-positive and further typed by RT-PCR. A total of 872 CVA16-positive samples were isolated and further sequenced to obtain the full-length VP1 sequence. Phylogenetic analysis was performed based on viral protein 1 gene (VP1). Results: In the first 25 weeks of 2024, reported cases of HFMD were 1.36 times higher than the mean rates of 2023. In 2024, CVA16 predominated at 75.42%, contrasting with the past etiological pattern in which the CVA6 was predominant with the detection rate ranging from 32.85 to 77.61% from 2019 to 2023. Phylogenetic analysis based on the VP1 gene revealed that the B1a and B1b subtypes co-circulated in Guangdong from 2018 to 2022. The B1c outbreak clade, detected in Guangdong in 2023, constituted 68.24% of the 148 strains of CVA16 collected in 2024, suggesting a subtype shift in the CVA16 virus. There were three specific amino acid variations (P3S, I235V, and T240A) in the VP1 sequence of B1c. Conclusions: The new emergence of the CVA16 B1c outbreak clade in Guangdong during 2023–2024 highlights the necessity for the enhanced surveillance of the virus evolution epidemiological dynamic in this region. Furthermore, it is imperative to closely monitor the etiological pattern changes in Hand, Foot, and Mouth Disease (HFMD) in other regions as well. Such vigilance will be instrumental in guiding future vaccination strategies for HFMD.

## 1. Introduction

Hand, Foot, and Mouth Disease (HFMD) is predominantly observed in children under five years old, particularly in the Asia–Pacific region [1]. It is typically characterized by fever and rash or herpes on the hands, feet, or mouth and potentially serious complications such as myocarditis and aseptic meningoencephalitis [2]. Mainland China has reported over one million cases and several hundred deaths due to HFMD annually since 2008 (http://www.nhc.gov.cn/, in Chinese, accessed on 30 June 2024), posing a persistent threat to health. Enterovirus A71 (EV-A71), Coxsackievirus A16 (CVA16), and Coxsackievirus A6 (CVA6) are considered the primary causative agents of HFMD [2].

CVA16 is a non-enveloped, single-stranded RNA virus, belonging to human enterovirus A (HEV-A) of the *genus Enterovirus* family *Picornaviridae*. The genome of CVA16 is approximately 7.4 kb in length, consisting of a single open reading frame (ORF) and two untranslated regions at the 5′ and 3′ ends (5′-UTR and 3′-UTR). The ORF encodes four structural proteins (VP1-VP4) and seven non-structural proteins (2A–2C, 3A–3D). VP1 is typically used for genotyping, categorizing CVA16 into genotypes A, B1, and B2, with genotype B1 further divided into subgenotypes B1a, B1b, and B1c [3]. Currently, B1a and B1b strains are the dominant clades of CVA16 in the world. Except for India, Malaysia, Russia, and France [4], the B1c genotype strains of CVA16 have been seldom reported worldwide. There are few reports of the B1c clades cases in China, all of which are sporadic cases. In 2022, multiple cases associated with B1c clades CVA16 were detected for the first time in Beijing [5], clearly indicating an upward trend in the B1c subgenotype.

The increasing prevalence of CVA16 outbreaks underscores the importance of maintaining vigilance in monitoring its epidemiological and genetic characteristics. In 2024, mainland China witnessed a significant upsurge in HFMD cases (http://www.nhc.gov.cn/, in Chinese, accessed on 30 June 2024). The official data revealed a 3.4-fold increase in HFMD cases in the first quarter of 2024 compared to 2023, highlighting the need for in-depth molecular epidemiological analyses. Therefore, we investigated the incidence and etiological patterns of HFMD in Guangdong, China, and examined the genetic variations in the emerged enterovirus genotypes.

## 2. Materials and Methods

### 2.1. HFMD Surveillance and Sample Collection

In 2008, Hand, Foot, and Mouth Disease (HFMD) was classified as a Class C notifiable disease. Guangdong Province established a monitoring system for HFMD, comprising 871 clinics and local Centers for Disease Control and Prevention (CDC) in 21 cities [6]. HFMD cases were identified based on the Ministry of Health diagnostic criteria: fever, mouth ulcers, blisters on the hands, and sometimes on the buttocks and knees. In the HFMD surveillance system, the CDCs of the 21 cities in Guangdong are responsible for sample collection from sentinel hospitals. A total of 40,673 clinical specimens were collected from suspected HFMD cases in Guangdong province from 2018 to 2024, including rectal swabs (n = 27,954), throat swabs (n = 6791), stool (n = 5923), cerebrospinal fluid (n= 3), and herpes fluid (n = 2). The clinical specimens are sent to the local CDC laboratories for detection of enterovirus (EV), EV-A71, CVA16, and CVA6 using real-time PCR (RT-PCR) [1]. Positive results are reported to the Guangdong Provincial CDC, with clinical specimens being submitted for further virus isolation and sequencing.

### 2.2. Virus Isolation and VP1 Sequencing

In line with the provincial HFMD surveillance program, CVA16-positive samples with a Ct value below 30 were prioritized for viral isolation. The number of viral isolates obtained from different regions or time periods was proportional to the corresponding number of reported positive cases. A total of 872 CVA16 viral strains were isolated on human rhab-domyosarcoma (RD) cells as described previously [7]. The isolates were harvested for viral RNA extraction and sequencing. Viral RNA is extracted from viral cultures using the QIAamp Mini Viral RNA Extraction Kit (Qiagen, Montgomery, MD, USA) according to the manufacturer’s instructions. For VP1 sequencing, PCR was performed with a QIAGEN OneStep RT-PCR Kit (Qiagen, USA) and the CVA16-specific primers CVA16-VP1-S (5′-ATTGGTGCTCCCACTACAGC-3′) and CVA16-VP1-A (5′-GCTGTCCTCCCACACAAGAT-3′) to amplify nucleotides 2446 to 3336 based on the reference strain CVA16/G-10 (prototype strain). PCR was performed with the following thermal profile: 50 °C 30 min, 95 °C 15 min, 36 cycles of 95 °C 30 s, 50 °C 40 s, and 72 °C 70 s, followed by a final extension at 72 °C 10 min. The PCR products were purified and sequenced using the BigDye Terminator v3.1 Cycle Sequencing Kit (Thermo Fisher Scientific, Waltham, MA, USA) on an Applied Biosystems 3500XL Analyzer (Thermo Fisher Scientific, Waltham, MA, USA). All CVA16 VP1 sequences generated in this study have been submitted to GenBank with accession numbers PQ872353–PQ872705.

### 2.3. Phylogenetic Analysis

We compared the 353 CVA16 VP1 sequences generated in this study with all publicly available 190 CVA16 VP1 sequences in GenBank that have known sampling dates. Multiple sequence alignment was performed using MAFFT version 7 [8], with CVA16/G-10 as the reference strain. The maximum likelihood (ML) tree was estimated using IQ-TREE version 2.3.6 [9]. Molecular-clock phylogeny was inferred by assessing the accumulation of genetic changes over time by root-to-tip regression in TreeTime version 0.11.4. Ancestral reconstruction and molecular clock homology were estimated using joint maximum likelihood analysis of TreeTime. The annotated data and metadata were compiled and exported in JSON format for visualization in the Auspice interactive phylodynamic tool [10] (https://auspice.us/, accessed on 30 June 2024).

## 3. Results

### 3.1. Incidence of HFMD Infection from 2018 to 2024

**Figure 1:**
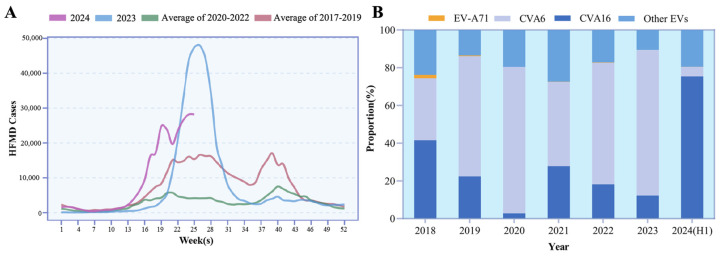
(**A**) Weekly reported HFMD cases in Guangdong, China (2023–Jun 2024). For comparative analysis, the average incidence rates of HFMD from the pre-COVID-19 era (2017–2019) and during the COVID-19 pandemic (2020–2022) are also charted; (**B**) etiological distribution of enteroviruses in HFMD cases (2018–Jun 2024 (H1)).

Guangdong has documented 241,844 HFMD cases within the first 25 weeks of 2024, marking a 1.36-fold increase over 2023 and significantly exceeding the mean rates of 2020–2022 and 2017–2019. The three to four weeks earlier and more rapid increase in HFMD cases suggests a potential shift in epidemiological pattern (Figure 1A). A total of 40,673 suspected samples of Hand, Foot, and Mouth Disease were collected in 2018–2024, of which 24,410 samples tested positive for EV (60.02%, 24,410/40,673). For EV genotype distribution, CVA6 accounted for 77.21%, being the predominant genotype in 2023 while CVA16 only 12.24%. In 2024, CVA16 predominated at 75.42%, eclipsing CVA6 at 5.00%. The detection of EV-A71 was minimal at 0.03%. The etiological pattern of HFMD was quite different from the surveillance result in the past five years for which the CVA6 was predominant with detection rate ranging from 32.85 to 77.61%. This contrasts with the past etiological pattern for which CVA6 has been predominant since 2019 (Figure 1B).

### 3.2. Phylogenetic Analysis

**Figure 2:**
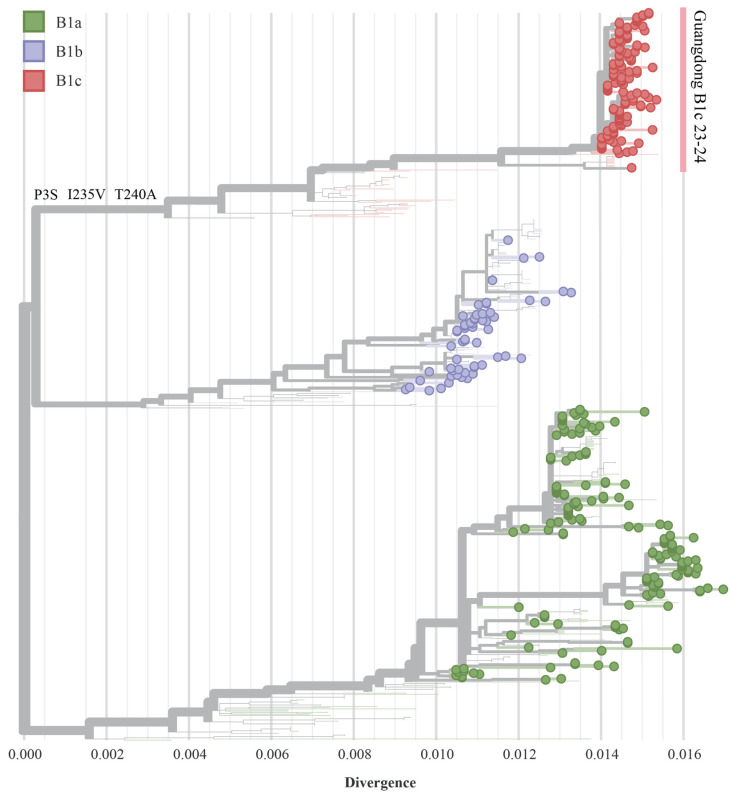
The maximum likelihood distance tree of CVA16 VP1 gene. The phylogenetic tree was constructed by including Guangdong CVA16 sequences (highlighted with dots) and all public available CVA16 sequences from 2018 to 2024 (not circled) to show the prevalence of CVA16 subgenotypes and their phylogenetic distances. Notable amino acid variations in the VP1 protein between the B1c subgenotype and B1a/B1b, are indicated on the B1c branch.

**Figure 3:**
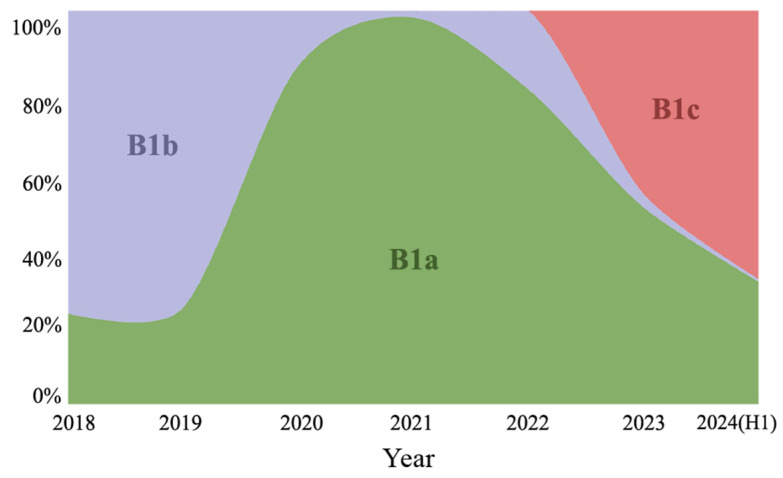
The temporal distribution of CVA16 subgenotypes (B1a, B1b and B1c) in Guangdong 2018–Jun 2024 (H1).

The phylogenetic analyses encompassing 353 Guangdong CVA16 strains from 2018 to 2024 and 190 globally available contemporary sequences, based on full-length VP1 genes (891 nucleotides, positions 2446–3336, relative to strain CVA16/G10). CVA16 were classified into different subgenotypes based on the sequence variability of the VP1 gene. CVA16 circulating in last five years could be classified into three different subgenotypes: B1a, B1b, and B1c, based on the diversity in the VP1 coding sequence of the structural protein (Figure 2). The subgenotypes B1a and B1b have been co-circulating in Guangdong, with the predominant strains changing over time. The proportion of B1a strains in Guangdong increased steadily after 2018, and B1b replaced B1a as the predominant subgenotype since 2020. The B1c subgenotype was observed in 2023 in Guangdong, with an increasing detection rate observed subsequently. In 2024, the B1c subgenotype accounted for 68.24% (101 of 148) of all sequenced CVA16 viruses in Guangdong (Figure 3). The similarity analysis revealed the following homologies: B1a and B1b had nucleotide and amino acid similarities of 85.50–94.70% and 96.05–100.00%, respectively; B1a and B1c showed similarities of 84.81–94.61% and 95.67–99.28%, respectively; and B1b and B1c exhibited similarities of 86.56–97.12% and 96.05–99.28%, respectively. The B1c subgenotype showed a high homology of 98.70–100.00% among the strains sequenced in 2024, indicating a high degree of similarity with the B1c strains in Guangdong, China. These B1c strains were genetically divergent (90.20–95.30% similarity in VP1 region) from the early circulation strains detected in France and other Asian regions. Without the timely molecular surveillance data from other regions, a potential origin of the new emerged B1c and its prevalence in other regions were largely unknown.

The amino acid mutation was frequently observed for subgenotypes of a specific enterovirus serotype. The ancestor reconstruction indicated the B1c subgenotype is distinguished from B1a and B1b by specific mutations at positions 3 (P3S), 235 (I235V) and 240 (T240A) in the VP1 protein (Figure 2 and Supplementary Figure S1).

## 4. Discussion

In this study, we analyzed HFMD-positive cases collected from Guangdong, China, from 2018 to 2024. Our study underscores a notable shift in the epidemiological and etiological patterns of HFMD in Guangdong in 2024. These surveillance data offers a new perspective on the prevention and control of HFMD, emphasizing the need for ongoing surveillance of this emerging subtypes in other regions, and to inform the development of a HFMD vaccine.

In this study, we analyzed the average incidence of HFMD between 2017 and the first half of 2024 and performed genotyping on 24,410 EV-positive samples. The number of HFMD cases in the first 25 weeks of 2024 experienced a significant surge, with a growth rate higher than the average in 2023, 2020–2022, and 2017–2019. This phenomenon points a possible change in the epidemiological characteristics of HFMD, especially with the rapid rise in cases in the early weeks of 2024. The dominance of CVA16 in 2024 presents a stark contrast to the period from 2019 to 2023, when CVA6 was the primary causative agent. The significant shift in the pathogen profile indicates that CVA16, particularly the B1c subtype, may have become the primary driving force behind the HFMD outbreaks in Guangdong. In addition, EV-A71 was detected in only 0.03% of the cases, a finding consistent with previous reports [1]. The gradually declining detection rate of EV-A71 may be related to the higher antibody levels in the population after vaccination. Therefore, it is essential to prioritize the development of vaccines specifically targeting CVA16 to better control the outbreaks of HFMD.

The EV structural protein VP1 is closely associated with the genotype classification of enteroviruses. Based on the complete VP1 gene sequences, CVA16 viruses could be classified into three main subgenotypes B1a, B1b, and B1c. The B1a clade was first identified in Japan in 1995 and became the major circulating strain of CVA16 in mainland China, Malaysia, and Thailand [6]. Since 2000, the B1b and B1a strains have co-circulated in China. The B1c clade was first detected in Malaysia in 2005 and subsequently detected in France and Japan causing an HFMD outbreak in India from 2009 to 2013 [11,12]. In recent years, B1c strains have occasionally been identified in Australia (2017) and the UK (2018) [5]. Reports on the B1c clade in China have also been limited to sporadic cases, including the case in Shanghai (2014) [13], Guangzhou (2018) [14], and Beijing (2016, 2022) [4,5]. In 2023, the B1c subtype was detected in Guangdong. In 2024, the B1c subtype accounted for 68.24% of CVA16, indicating a genotypic shift in CVA16 in Guangdong. Due to the lack of timely molecular surveillance data from other regions, its potential origin and spread in other areas remain largely unknown. Alignment with B1a and B1b, the B1c VP1 coding region has three amino acid mutations: P3S, I235V, and T240A. These three mutations have also been reported in recent studies on CVA16 in China [4,5]. Therefore, it remains to be investigated whether the amino acid variations at the VP1 positions of CVA16 identified in this study have led to changes in the antigenicity of the circulating CVA16 strains. Our ancestral reconstruction analysis show there is no amino acid change between the new emerged B1c in Guangdong and other B1c strains circulating in the other regions. Further study should be employed to find whether there are mutations out of VP1 resulting in any viral adaptation of the outbreak of B1c strains in Guangdong 2024.

Our primary surveillance showed the surge of the HFMD epidemic in Guangdong, China, 2024, was associated with the emergence of the antigenically distinct B1c subtype of CVA16. Currently, there are several multivalent vaccines related to HFMD-associated enteroviruses in the clinical trial phase. These multivalent vaccines all contain CVA16 and are developed based on the B1a and B1b subtypes that were prevalent domestically in the early stages. The antigenic differences between the subgenotypes necessitate a reevaluation of the existing vaccines and underscore the need for vaccines that can elicit a robust and cross-protective immune response. The limited molecular surveillance of HFMD in regions beyond mainland China raises concerns regarding the actual prevalence of the emerging CVA16 B1c subgenotype, potentially understating its global impact.

## Data Availability

All sequences generated in this study have been submitted to the GenBank database (PQ872353–PQ872705). The json files for VP1 phylogenetic trees are available at zenodo with record 14771486 (https://zenodo.org/records/14771486, accessed on 1 January 2025).

## Supplementary Materials

The following supporting information can be downloaded at: https://www.mdpi.com/article/10.3390/v17020219/s1, Figure S1: Sequence logo of amino acid mutations specific to the B1a, B1b, and B1c clades.
