# Supplementary figures and images for: The Emergence of Coxsackievirus A16 Subgenotype B1c: A Key Driver of the Hand, Foot, and Mouth Disease Epidemic in Guangdong, China
Source: Viruses. 2025 Feb 3;17(2):219. doi: 10.3390/v17020219 (PMC11860377; doi:10.3390/v17020219)

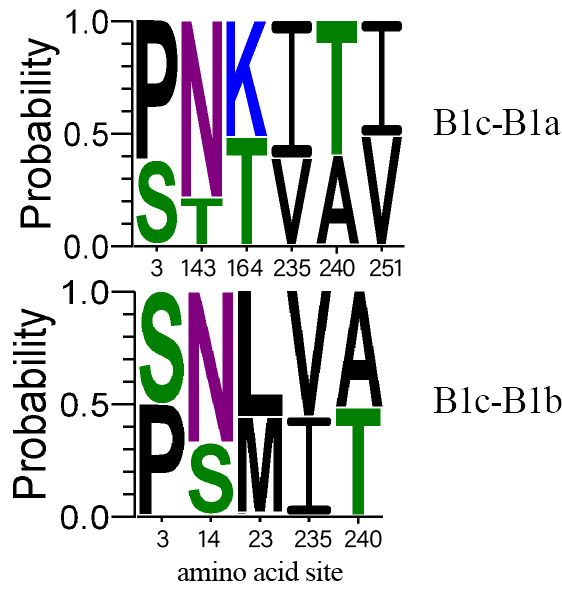

Supplement: Supplementary file 1 [file viruses-17-00219-s001.zip › viruses-3404143-supplementary.png]
